# Cellular Functions of Genetically Imprinted Genes in Human and Mouse as Annotated in the Gene Ontology

**DOI:** 10.1371/journal.pone.0050285

**Published:** 2012-11-30

**Authors:** Mohamed Hamed, Siba Ismael, Martina Paulsen, Volkhard Helms

**Affiliations:** 1 Center for Bioinformatics, Saarland University, Saarbrücken, Germany; 2 Department of Genetics, Saarland University, Saarbrücken, Germany; University of Bonn, Institut of experimental hematology and transfusion medicine, Germany

## Abstract

By analyzing the cellular functions of genetically imprinted genes as annotated in the Gene Ontology for human and mouse, we found that imprinted genes are often involved in developmental, transport and regulatory processes. In the human, paternally expressed genes are enriched in GO terms related to the development of organs and of anatomical structures. In the mouse, maternally expressed genes regulate cation transport as well as G-protein signaling processes. Furthermore, we investigated if imprinted genes are regulated by common transcription factors. We identified 25 TF families that showed an enrichment of binding sites in the set of imprinted genes in human and 40 TF families in mouse. In general, maternally and paternally expressed genes are not regulated by different transcription factors. The genes *Nnat*, *Klf14*, *Blcap*, *Gnas* and *Ube3a* contribute most to the enrichment of TF families. In the mouse, genes that are maternally expressed in placenta are enriched for AP1 binding sites. In the human, we found that these genes possessed binding sites for both, AP1 and SP1.

## Introduction

Genomic imprinting is an epigenetic phenomenon observed in eutherian mammals. For the large majority of autosomal genes, the two parental copies are both either transcribed or silent. However, in a small group of genes one copy is turned off in a parent-of-origin specific manner thereby resulting in monoallelic expression. These genes are called ‘imprinted’ because the silenced copy of the gene is epigenetically marked or imprinted in either the egg or the sperm [1].

Imprinted genes play important roles in development and growth both pre- and postnatally by acting in fetal and placental tissues [2]. Interestingly, there appears to exist a general pattern whereby maternally expressed genes tend to limit embryonic growth and paternally expressed genes tend to promote growth. A model case for this striking scenario is the antagonistic action of *Igf2* and *Igf2r* in mouse. Deletion of the paternally expressed *Igf2* gene results in intrauterine growth restriction. On the other hand, deletion of the maternally expressed gene *Igf2r*, results in overgrowth [3].

The observation that maternally and paternally expressed genes apparently act as antagonists has inspired several evolutionary theories that aim to explain the origin of genetic imprinting under the process of ‘natural selection’ [2]. The most scientifically accepted theory is currently the kinship theory [4] and [5]. Briefly, this theory suggests that in polygamous mammalian species, silencing of maternally derived growth inhibiting genes results in increased growth of the embryo. This is associated with an increased nutritional demand and thereby with an exploitation of maternal resources at the cost of future off-spring that might be fathered by a different male.

The evolution of a gene regulatory mechanism that silences preferentially one parental allele of a gene implies that paternally and maternally expressed genes experience different selective pressures during evolution. This assumption is supported by the finding that the two groups reveal different patterns of sequence conservation. Whereas the protein-encoding DNA sequences of paternally expressed genes are well conserved among different mammalian species, maternally expressed genes are much more divergent [6]. Whether paternally and maternally expressed genes differ also in molecular functions and gene regulation is a question that has not yet been investigated in detail. Many studies showed that imprinted genes are not only important during embryonic development but possess also postnatal functions. Hence, kinship theory with its focus on prenatal development might explain some but not all aspects of the evolution of genomic imprinting.

During postnatal development, genomic imprinting affects endocrinal networks, energy metabolism, and behavior. Prominent examples for the functions of imprinted genes in endocrinal pathways are the imprinted transcripts of the *Gnas* locus. In the human, genetic and epigenetic aberrations in this region are associated with Albright hereditary osteodystrophy and pseudohypoparathyroidism type 1A or 1B [7]. Behavioral abnormalities have been observed in human imprinting disorders and in various mouse models in which imprinted genes have been mutated. For example, the obesity of Prader-Willi-syndrome patients is, at least in parts, a result of an impaired eating behavior. Knock-out studies in mouse showed that the two paternally expressed *Peg1* and *Peg3* genes have a clear behavioral phenotype [8]. Females that inherit a null allele for these genes from their fathers behaved ‘deficiently’ with respect to maternal care behavior including placentophagy and nest-building as well as pup gathering.

As the phenomenon of genomic imprinting is an important evolutionary facet of mammals with placentas, it is of great interest to identify which sorts of cellular and developmental processes of developing and/or mature organisms are subject to control by imprinted genes. We aimed in this study at characterizing the cellular roles of imprinted genes in an unbiased, data-driven approach. For this, we used the gene annotations of the Gene Ontology (GO) that consists of three structured and controlled vocabularies for the biological processes, cellular components, and molecular functions associated with particular genes. As it is of particular interest to analyze which of these functions are controlled by the sets of maternally and paternally expressed genes, we have also separately analyzed the enrichment of GO terms in these two groups.

## Methods

### Gene Selection

Imprinted genes of human and mouse were downloaded from the Catalogue of Imprinted Genes and Parent-of-origin Effects in Humans and Animals (IGC) [9] and [2]. The catalogue encompasses genes that were described as being imprinted in literature. As the related experiments were done in many different labs, the experimental procedures differed considerably. After reading the original publications, we manually selected 64 imprinted genes that are imprinted without doubt in at least one of the two species, see table S1. For the gene *C15orf2*, the expressed allele is unknown since there is no information on the parental origin of the alleles. *Copg2*, and *Zim2* are paternally expressed in the human, but maternally expressed in the mouse. *Grb10* exhibits isoform-specific imprinting effects, i.e. there are paternally expressed and maternally expressed isoforms. The other 60 genes have been experimentally classified into paternally and maternally expressed alleles in two equal halves. 25 genes are imprinted in both species, for the remaining imprinted expression was proven only for one of the two species. As control group for the human (mouse) imprinted genes we used all human (mouse) genes that are annotated in the Gene Ontology.

### Functional Enrichment Analysis

For analyzing significantly enriched functional categories, we used the functional annotation tool available in the Database for Annotation, Visualization and Integrated Discovery (DAVID) [10]. We determined which GO categories are statistically overrepresented in different sets of genes. Enrichment was evaluated through the Fisher Exact test using a significance level or p-value threshold of 0.05. We suspected that some functional categories with a high statistical significance may show over-representation even when annotated only to a single gene. In that case, it would not be clear if this function is related to monoallelic expression of the gene in certain tissues, or when it is biallelically expressed in other tissues. Therefore we required that each GO term considered here is annotated to at least two human (mouse) genes.

For the most specific GO terms, we ran the same enrichment analysis procedures by using the biological process GO_FAT database instead of using the general GO knowledgebase. GO_FAT is a subset of the full set of GO terms that was established by the DAVID team so that the broadest terms should not overshadow more specific terms. The smaller the p-value, the more enriched is the corresponding GO term in the group of imprinted genes with respect to all human or mouse genes. The map enrichment plugin in Cytoscape [11] was used to visualize the overrepresented functional terms and display the overlapping functional sets.

### Gene Functional clustering

Clustering and grouping of the imprinted genes were performed using the DAVID gene functional classification tool. This tool employs a set of fuzzy clustering techniques to classify input genes into functionally related gene groups (or classes). This is done on the basis of the co-occurrence of annotation terms by generating a gene-to-gene similarity matrix based on shared functional annotation. This switches the functional annotation analysis from a gene-centric analysis to a biological module-centric analysis [10]. The similarity threshold was set to the minimum similarity threshold of 0.3 suggested by the DAVID consortium. This is then the minimum value to be considered by the similarity-matching algorithm as biologically significant. Also, we set the minimum gene number in a seeding group to 2. This would be the minimum size of each cluster in the final results. All remaining parameters were kept to their default values. The results of the functional classification tool are visualized as heat maps to show the corresponding gene-annotation association across the clustered genes.

### Transcription Factor Target Enrichment

The web-based gene set analysis toolkit WebGestalt [12] was used to analyze the targets of transcription factors (TFs), see tables S7 and S8. This tool incorporates information from different public resources such as NCBI Gene, GO, KEGG and MsigDB (http://bioinfo.vanderbilt.edu/webgestalt/). Using the TF target analysis tool implemented in WebGestalt, we analyzed whether a set of genes is significantly enriched with TF targets (TFT). TFT's are specific sets of genes that share a common TF-binding site defined in the TRANSFAC database [13]. TFT's are collected in the Molecular signature Database (MsigDB) [14] and are retrieved by WebGestalt upon analysis request. The examined promoter region has the size of −2 kb, +2 kb around the transcription start site. Then enrichment was evaluated through the hypergeometric test using the 10 most enriched terms with maximum significance level or p-value of 0.05. As we are testing multiple TFT families at the same time, the p values need to be adjusted for the effects of multiple testing. For this we applied the sequential Bonferroni type procedure method proposed by [15]. We only considered enrichment of TFT families that were annotated for at least two genes. Finally, the results of the TFT enrichment analysis were visualized as heat maps to identify the common principles and differences of the enriched TF targets across the corresponding imprinted genes. This was done using the statistical language R [16].

## Results

In this study we addressed the question whether imprinted genes as a group fulfill specific functions in mammalian organisms. For this, we tested if specific GO terms are overrepresented in the group of imprinted genes in comparison to all genes in the human or mouse genome. Of the 41 selected human imprinted genes, 38 are annotated in the GO database that contains in total 14116 human genes. In contrast, all 48 mouse imprinted genes are among the 14219 annotated mouse genes. One should note, though, that many genes are represented by more than one transcript in the GO database.

### Imprinted genes are involved in developmental, transport and regulatory functions

First, we analyzed which terms of the Gene Ontology are enriched in the full set of all imprinted genes when compared to the set of all human genes or all mouse genes. We concentrate in this analysis on GO terms that are shared by at least 2 different imprinted genes. In this way, we assume to emphasize those cellular functions that relate to the controlled mono-allelic expression of the set of genes studied here. The terms of the GO database are organized in a tree-like structure where a few general terms such as *developmental process* are linked to numerous more specific terms on the next hierarchical level. Terms that showed an overrepresentation of imprinted genes in both human and mouse with p-values below 0.05 are listed in supplemental tables S2 and S3.

In the human, the term *system development* is the term with the lowest p-value. This term is associated with 15 out of the 38 human imprinted genes. This corresponds to a 2.6 fold enrichment in comparison to the annotation frequency in the group of all genes. *Cellular processes* is the term which is associated with the largest number of imprinted genes in the human: 32 imprinted genes (84.2% of all imprinted genes) are associated with this term, whereas this is only the case for 74.6% of all genes. For comparison, the imprinted genes in mouse showed a narrower range of 1.8 and 2 fold enrichment for these two broad terms, and only for *system development* the p-value is below 0.05. As shown in Table 1, only the five generic GO terms, *multicellular organismal development*, *developmental process*, *neuron development*, *system development*, and *anatomical structure development* appear in both species with close to 2-fold enrichment (p<0.05, Fisher exact test). Only *neuron development* is 5-fold enriched.

**Table 1:** Conserved functional classes in imprinted genes in human (green) and mouse (brown) at a p-value of 0.05.

Term	Species	Count	Percentage	Fold Enrichment	−Log (p-value)
GO:0007275 ∼multicellular organismal development	Human	16	42.1	IIIIIIIIIIIIIIIIIIIIII 2.3	IIIIIIIIIIIIIIIIIIIIIIIIIIII 2.8
	Mouse	14	29.2	IIIIIIIIIIIIIIIIIII 1.9	IIIIIIIIIIIIIIIII 1.8
GO:0032502 ∼developmental process	Human	17	44.7	IIIIIIIIIIIIIIIIIIIII 2.2	IIIIIIIIIIIIIIIIIIIIIIIIIIIII 2.9
	Mouse	15	31.3	IIIIIIIIIIIIIIIIIII 1.9	IIIIIIIIIIIIIIIIII 1.8
GO:0048666 ∼neuron development	Human	4	10.5	IIIIIIIIIIIIIIIIIIIIIIIIIIIIIIIIIIIIIIIIIIIIIII 4.8	IIIIIIIIIIIII 1.3
	Mouse	4	8.3	IIIIIIIIIIIIIIIIIIIIIIIIIIIIIIIIIIIIIIIIIIIIIII 4.8	IIIIIIIIIIIII 1.3
GO:0048731 ∼system development	Human	15	39.5	IIIIIIIIIIIIIIIIIIIIIIIIII 2.6	IIIIIIIIIIIIIIIIIIIIIIIIIIIIIIII 3.3
	Mouse	12	25.0	IIIIIIIIIIIIIIIIIIII 2.1	IIIIIIIIIIIIIIII 1.7
GO:0048856 ∼anatomical structure development	Human	15	39.5	IIIIIIIIIIIIIIIIIIIIIII 2.4	IIIIIIIIIIIIIIIIIIIIIIIIIIII 2.9
	Mouse	12	25.0	IIIIIIIIIIIIIIIIIII 1.9	IIIIIIIIIIIIII 1.5

**Figure 1:**
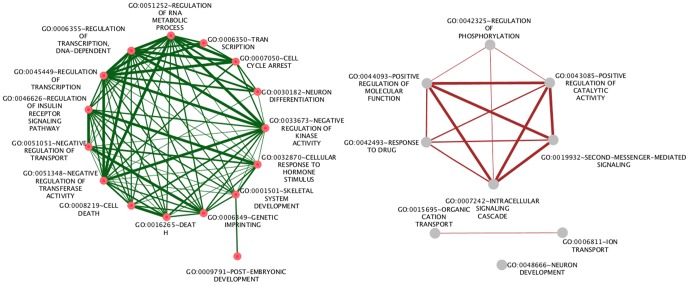
The most specific enriched GO terms of biological functions for the full set of imprinted genes in human (green) and mouse (brown). Nodes represent the enriched Go terms and the thickness of the interconnected links corresponds to the number of shared genes.

As terms such as *system development* and *cellular processes* are rather general terms, we subsequently analyzed the enrichment of terms in the GO_FAT section of the DAVID database. This manually curated section contains only terms that are related to rather specific functions. As shown in Figure 1, among the enriched specific terms in human and mouse, some are linked to neuron development and differentiation and are intimately related with the *CDKN1C* and *NDN* genes. Interestingly, the terms *regulation of RNA metabolic process*, *regulation of transcription*, *DNA-dependent*, and *regulation of transcription* are the terms that are associated with the largest numbers of human imprinted genes (28.9, 28.9 and 34.2%, respectively). Moreover, around 8.5% and 10.5% of the examined mouse imprinted genes are involved in the regulation process of phosphorylation and positive regulation of molecular function, respectively. This group includes the imprinted genes *Igf2*, *Ins2*, *Kcnq1*, *Htr2a*, *Grb10*, *Ndn*, *Tp73*, *Impact*, *Cdkn1c*, *Zim2*, and *Plagl1*.

The two GO terms *Regulation of RNA metabolic process* and the daughter node *Regulation of transcription*, *DNA-dependent* are associated with processes involved in the role of RNA synthesis regulation. Some of the encoded proteins are tumor proteins; others are inhibitors of the cell cycle, thus inhibiting division. It is also worth mentioning that the functional term *regulation of gene expression by genetic imprinting* (this is abbreviated to ‘*genetic imprinting*’ in the DAVID database) is over-represented as well and is associated with the genes *INS*, *IGF2*, and *KCNQ1* (Note: *INS* and *IGF2* are being interpreted by DAVID as a single locus that includes two alternatively spliced read-through transcript variants and align to the *INS* gene in the 5′ region and to the *IGF2* gene in the 3′ region). These functional associations of *IGF2* and *KCNQ1* rely on publications reporting how a differentially methylated region in *KCNQ1* controls imprinted expression of other genes in the neighborhood [17] and about epigenetic abnormalities in the *IGF2/H19* region of Beckwith-Wiedemann syndrome patients [18]. Note that being associated with the GO term *regulation of gene expression by genetic imprinting* therefore does not refer to the “property” of the respective gene to be an imprinted gene itself but indeed whether it exerts regulatory function on other genes via genetic imprinting. Consequently, the insulator protein CTCF and the DNA methyltransferase DNMT3A are associated with this term as well.

Some functions related to transport are enriched and associated with both human and mouse imprinted genes. For instance, the Growth factor receptor-bound protein 10 (*GRB10*) is involved in the *Negative regulation of transport*. This gene interacts with insulin receptors and insulin-like growth-factor receptors [19]. Overexpression of some isoforms of *GRB10* inhibits tyrosine kinase activity and results in growth suppression, e.g. by suppressing glucose import [20]. The two enriched GO terms *Organic cation transport* and *Ion transport* describe the regulation of the directed movement of organic cations into, out of or within a cell, or between cells, by means of some agent such as a transporter or pore. The associated mouse imprinted genes *Slc22a2* and *Slc22a3* are polyspecific organic cation transporters in the liver, kidney, intestine, and other organs.

**Figure 2:**
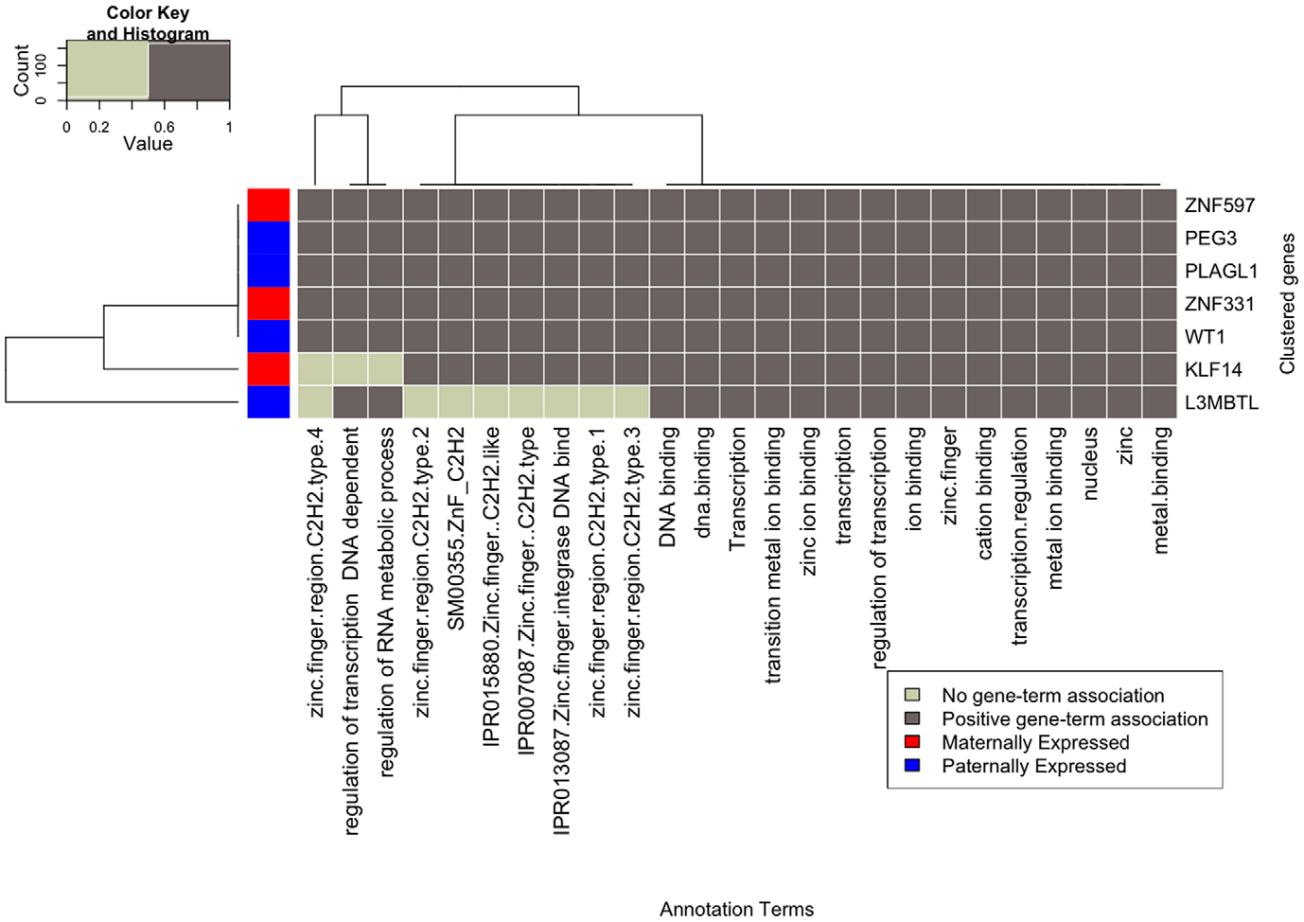
Functionally related imprinted genes in human. The heat map view shows the gene-term association for those genes that share a high number of associated GO terms. Marked in red on the left side are maternally expressed genes; marked in blue are paternally expressed genes.

**Figure 3:**
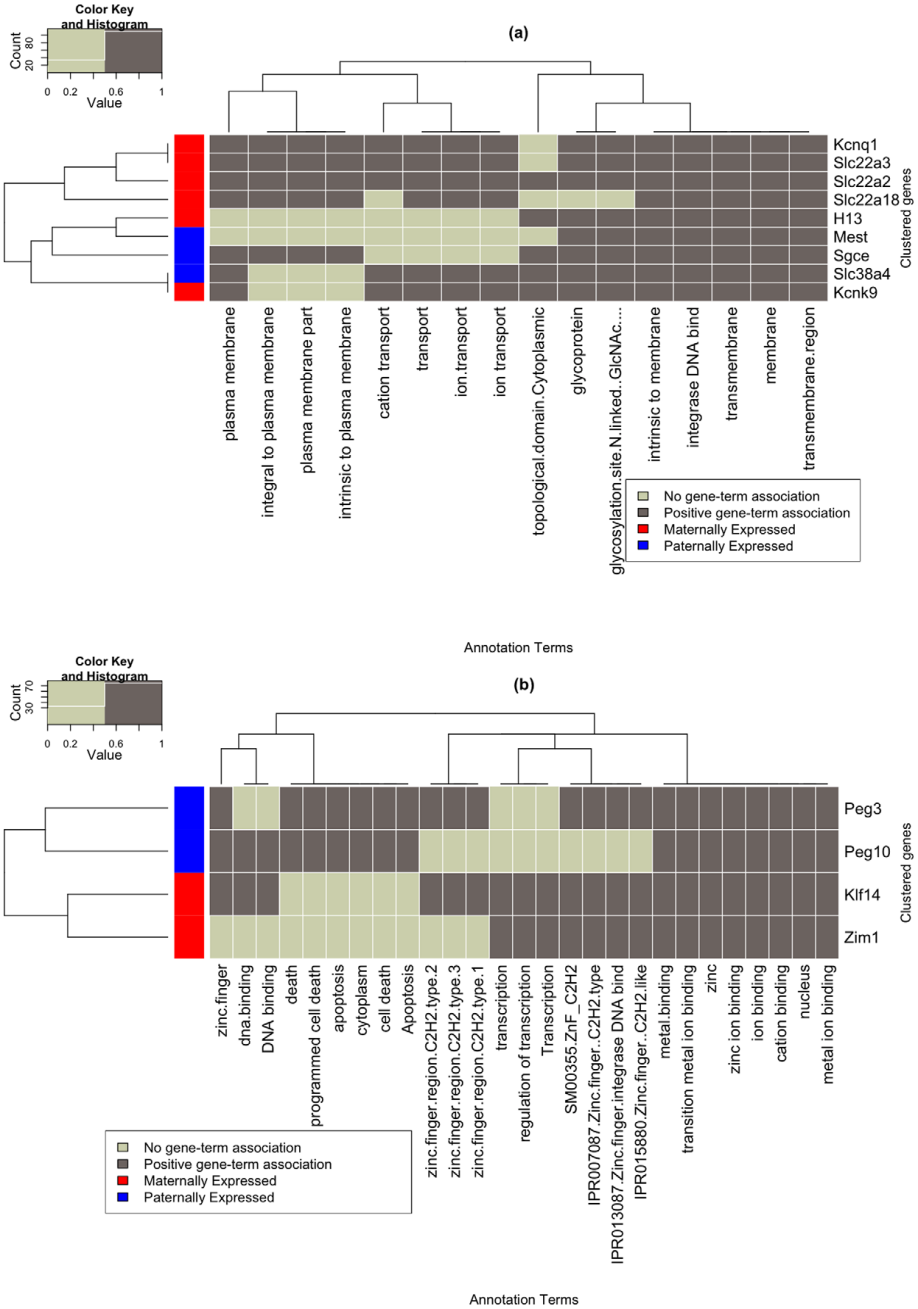
Functionally related imprinted genes in mouse. Heat maps showing the gene-term association for the first and second gene clusters in Mouse. Marked in red on the left side are maternally expressed genes; marked in blue are paternally expressed genes.

Grouping genes based on shared GO terms can highlight functional similarities of different genes. For this, clustering algorithms were applied to a gene-to-gene similarity matrix and imprinted genes were classified into highly related groups (see methods). We identified one gene cluster in the human and two clusters in the mouse. The only discovered cluster in human resembles the second cluster in mouse and encompasses zinc finger protein genes such as *PEG3*, *ZNF597* and *ZNF331*. Its members have a strong association with regulatory and transcriptional tasks (Figure 2). For mouse, the first cluster contains mostly genes that encode proteins that are involved in transport processes (Figure 3a). As mentioned, the second group consists mostly of zinc finger protein genes similar to the human one (Figure 3b).

### Maternally expressed genes dominate the role of imprinted genes in transport and gene regulation

In previous studies [6], we showed that maternally and paternally expressed genes differ in the level of conservation of their DNA sequences. For this reason, we analyzed whether maternally and paternally expressed genes differ also in their biological and molecular functions. For the 19 maternally expressed genes in human, only 3 broad functional terms were found to be enriched, *nervous system development*, *organ morphogenesis*, and *positive regulation of osteoblast differentiation*. For the last GO term, the maternally expressed genes even showed a 59.4-fold enrichment (see table S4) although only two imprinted genes (*DLX5* and *GNAS*) are associated with this term. Therefore, the enormous enrichment likely reflects that *positive regulation of osteoblast* is so far associated with very few genes in the full genome.

**Figure 4:**
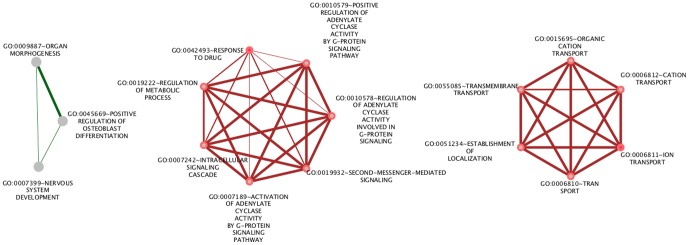
The enriched GO terms of biological functions for the maternally expressed genes in human (green) and mouse (brown). Nodes represent the enriched Go terms and the thickness of the interconnected links corresponds to the number of shared genes.

In mouse, 24 genes are classified as maternally expressed. We found that 14 biological functions are significantly associated with these genes. These 14 terms (table S4) are dominated by a group of relatively unspecific terms related to transport processes such as *organic cation transport*, *transmembrane transport*, *ion transport* and *organic cation transport*. Therefore, not surprisingly, the five maternally expressed genes *Kcnk9*, *Kcnq1*, *Slca22a2*, *Slca22a3* and *Slca22a18* form a gene cluster that is associated with the same transport-related GO terms. The second gene cluster is formed by TF genes including the maternally expressed genes *Klf4* and *Zim1* (Figure 4).

### Only few paternally expressed genes in human possess similar functions

**Figure 5:**
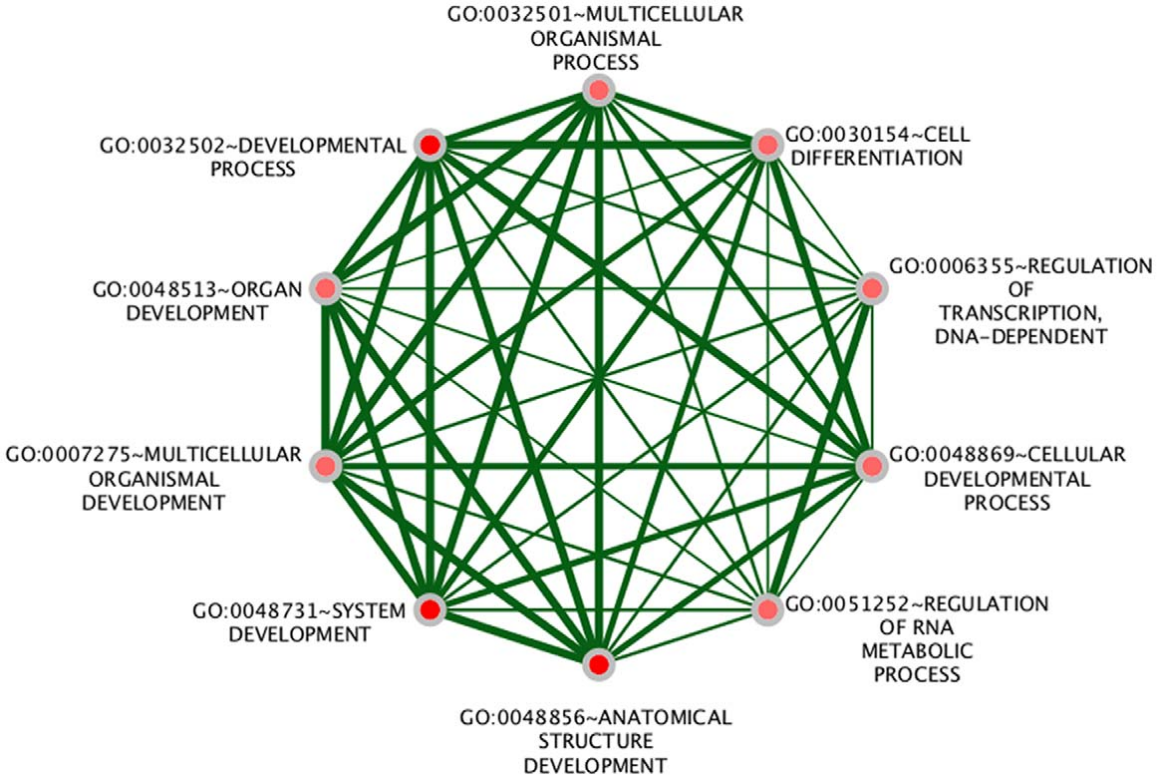
The enriched GO terms of biological functions for the paternally expressed genes in human. Nodes represent the enriched Go terms and the thickness of the interconnected links corresponds to the number of shared genes.

The 17 paternally expressed genes in human are associated with fewer over-represented GO terms (p<0.05) than the maternally expressed genes. Most of them were already present in the over-represented terms for all imprinted genes (Figure 5 and Table S5). Thus we examined these genes on the basis of the GO_FAT knowledge base that contains more specific terms. Only two terms, i.e. *regulation of transcription*, *DNA-dependent* and *regulation of RNA metabolic process* are enriched for paternally expressed genes. Both terms are associated with the genes *PLAGL1*, *L3MBTL*, *IGF2*, *WT1*, *ZIM2*, and *PEG3* (table S6). Hence, both maternally and paternally expressed genes contain prominent groups of genes that have regulatory roles. Paternally expressed genes in mouse did not show any significant enrichment.

### Enrichment analysis for the transcription factor targets

Mammalian genes are usually controlled by combinations of different TFs that bind to distinct binding sites in regulatory regions such as the promoters of genes. We were interested in the questions which TFs regulate imprinted genes and if paternally and maternally expressed genes can be distinguished by their TFs. For addressing these questions we applied a similar enrichment analysis (see Methods) to investigate whether binding sites for distinct TFs are enriched in the promoter regions of imprinted genes. This analysis was based on a database of TF targets named Molecular signature Database (MsigDB) [14]. This data set consists of sets of genes, the so-called TF targets families, that share binding sites for the same transcription factor families.

**Figure 6:**
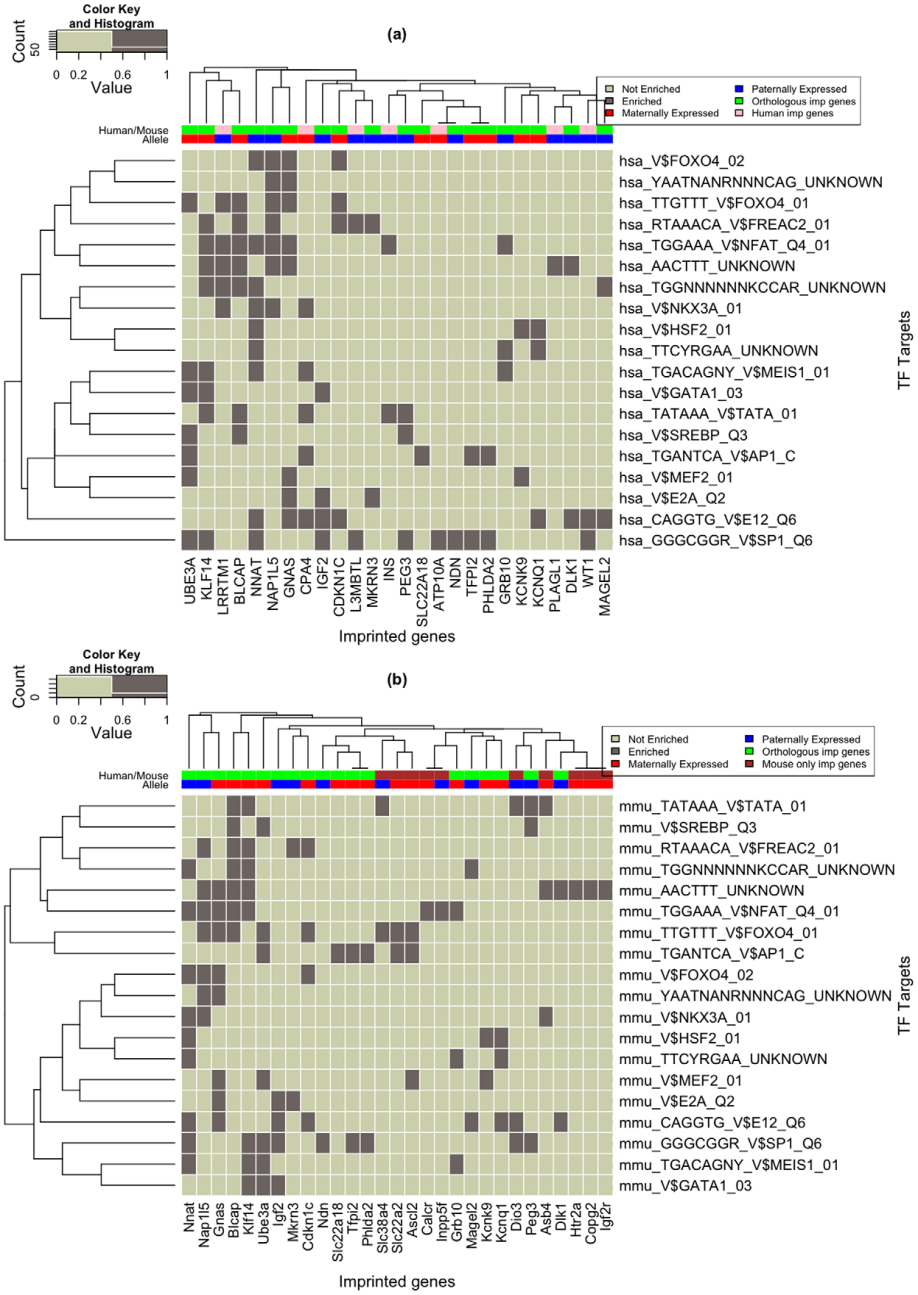
Conserved transcription factors in the full set of imprinted genes in human (a) and mouse (b) at p-value of 0.01. Marked in red and blue in the top line are the maternally, paternally expressed genes, respectively. Genes that are imprinted in both species are marked in green. Pink are the genes shown to be imprinted only in human, and brown are the genes shown to be imprinted only in mouse.

In total, we identified 25 TF families that showed an enrichment of binding sites in the set of imprinted genes in human (p<0.01, hyper-geometric test, see Methods) (Table S7). The associations between these families and the corresponding genes are shown in Figure S1 (a) together with the expressed allele type. For mouse, binding sites for 40 TF families are enriched in imprinted genes at the same significance level of 0.01, see Figure S1 (b) and table S8. 19 transcription factor families possess binding sites that are enriched in the imprinted genes in both species (Figure 6). In species, *Nnat*, *Klf14*, *Blcap*, *Gnas*, and *Ube3a* are the genes that contribute most to the enrichment of transcription factor binding sites.

Figures 6 shows that in mouse and human, imprinted genes form similar, but not identical, clusters of genes that are regulated by the same transcription factor families. For example, the potassium channel genes *Kcnq1* and *Kcnk9* show an enrichment of heat shock factor 2 (HSF2) binding sites in human and mouse. Similarly, genes that are maternally expressed in placenta, such as *Slc22a18*, *Tfip2*, and *Phlda2*, cluster together in both species. In the mouse, this cluster is characterized by an enrichment of AP1 binding sites, whereas the prominent feature of the human gene cluster is a combination of AP1 and SP1 sites. Finally, Figure 6 illustrates clearly that paternally and maternally expressed genes do not cluster apart. This is also not the case if species-specifically enriched transcription factor binding sites are included (data not shown). Hence, paternally and maternally expressed genes are apparently not regulated by distinct combinations of TFs. and cannot be distinguished on a general level.

## Discussion

This study analyzed enriched functional annotations of genetically imprinted genes based on the “biological process” tree of the Gene Ontology. In their seminal review [21], Tycko and Morrison concluded that the group of imprinted genes is predominantly involved in controlling growth and neurobehavioral traits. Tycko and Morrison pointed out that the numbers of paternally and maternally expressed genes related to growth are almost identical. On the other hand, only one maternally expressed gene (*UBE3A*) was linked to behavioral functions, in contrast to three paternally expressed genes (*SGCE*, *NDN*, *PWCR1*), as well as the paternally expressed genes *PEG1 (MEST)* and *PEG3* that were related both to growth and behavior. Thus, Tycko and Morrison argued that imprinting effects due to either maternally or paternally expressed genes are related to growth whereas behavioral functions are mostly controlled by paternally expressed genes. However, at the present stage, it is unclear if imprinted genes act indeed in the control of behavior, or if the observed behavioral abnormalities in mutant mice are caused by an impaired development of neurons and brain structures.

Our study did reveal an association of imprinted genes with developmental processes such as organ development in human and mouse. This indicates that these genes function indeed during embryogenesis, but they are not necessarily growth regulating genes. The terms that are related to development in human as well as in mouse are associated with 25% to 44.7% of all imprinted genes in the respective species. Hence, a large proportion of imprinted genes contribute to developmental processes. Imprinted genes are also associated with GO terms that are related to neuronal development. Interestingly, neuronal development is apparently not a function that is restricted to paternally expressed genes. Furthermore, in comparison to developmental functions only a rather small number of imprinted genes (7 genes) show a functional association to the nervous system [22].

Several publications have pointed out that imprinted genes play roles in placenta morphology and function. We do not observe a specific association with GO terms that are specifically related to the placenta. Hence, at the first glance our results do not support specific roles in the placenta. However, one should note that many genes that show an expression bias towards the maternal allele in the placenta but not in the embryo have been excluded from this analysis. This was done since it is still under discussion if such biases might be mostly caused by sample contamination with maternal tissue [23].

When paternally and maternally expressed genes are analyzed separately, mouse and human show clearly different associations. In the human, several maternally expressed genes (*DLX5*, *GNAS*, *TP73*, *PHLDA2*, *CDKN1C*, *PPP1R9A*, *UBE3A*) are associated with *organ morphogenesis*, and more particularly with *nervous system development* and *oesteoblast differentiation*. In the mouse, maternally expressed genes form two functional networks that are clearly separated. One is related to transport processes, and includes carrier proteins and channel proteins. Especially transport processes that are a key feature of placenta function are specifically associated with maternally expressed genes in the mouse. The second network consists of terms related to G protein signaling. This network is clearly dominated by *CALCR* and *SLC22A18*. For the paternally expressed genes, a functional network is only found in the human. This network consists mostly of terms associated with development, and a few terms that are related to gene regulation. Interestingly, several imprinted genes that encode transcription factors (*PLAGL1*, *L3MBTL*, *WT1*, *ZIM2*, *PEG3*) seem to be key players in this network. Nevertheless, also among the maternally expressed genes are genes that regulate transcription. Thus, regulatory functions are not an exclusive feature of paternally expressed genes.

The differences between mouse and human can in parts be explained by evolutionary divergence. For example, human and mouse placentae show pronounced differences in morphology. In a previous publication we have shown that especially maternally expressed genes experienced an accelerated sequence divergence that were less prominent in the human [6]. These differences in molecular evolution might be associated with functional differences.

In this context we will briefly consider possible biases in the results obtained. The annotations stored in the Gene Ontology of course only represent a fraction of all knowledge in the original scientific literature and it is impossible to estimate how much we still don't know. It is quite likely that the GO gives a more complete picture about the cellular functions of genes that have been studied intensely compared to the average gene. It is furthermore possible that some of the known imprinted genes such as *IGF2* belong to the group of intensely studied genes so that their cellular functions are known to a larger extent than those of less well studied genes and when compared to the average bi-allelically expressed gene. In agreement with this idea, we found that the three well-known genes *IGF2*, *INS*, and *GRB10* (out of 30) tended to dominate the functional enrichments in the group of paternally expressed genes. In contrast, the enrichments in the group of all imprinted genes were stable even when we removed the well-known genes *IGF2*, *INS*, and *GRB10*.

When grouping the imprinted genes by enriched GO annotations found for at least two genes, we applied the lowest recommended threshold value of 0.3. In future, when more complete functional associations will be available, it remains to be tested whether a higher, more cautious threshold would be advantageous. We found that when applied to the currently available data, this threshold gave a good compromise between coverage and specificity of the obtained results.

In the second part of the study, we were interested in the question if functionally related gene groups such as the prominent groups of transcription factors, and transport related proteins, are co-regulated by similar sets of transcription factor families. This is obviously not the case. Interestingly, also maternally and paternally expressed genes are not regulated by distinct sets of transcription factor families. In general, a few genes, i.e. *UBE3A*, *KLF14*, *BLCAP*, *NAP1L5*, *NNAT*, and *GNAS*, show an over-proportional enrichment of distinct transcription factor binding sites. Interestingly, these genes possess rather diverse functions. For example, *UBE3A* seems to act in neuronal development, whereas *GNAS* acts mostly in endocrinal pathways.

Although imprinted genes appear to be regulated by similar sets of transcription factors in mouse and human, it is difficult to identify a typical transcription factor that regulates imprinted genes. The most prominent factor appears to be SP1. This rather ubiquitous factor might be responsible for the broad tissue spectrum of imprinted genes [24]. On the other hand SP1 deficiency is to some extent associated with placental defects and impaired ossification, that are typical features of defects in imprinting [25].

Varrault and co-workers have recently identified a network of coregulated imprinted genes involving the genes *Plagl1*, *Gtl2*, *H19*, *Mest*, *Dlk1*, *Peg3*, *Grb10*, *Igf2*, *Igf2r*, *Dcn*, *Gnas*, *Gatm*, *Ndn*, *Cdkn1c* and *Slc33a4* [26]. According to Fig. 6(b), E12 regulates four genes from this list (*Dlk1*, *Cdkn1c*, *Igf2* and *Gnas*); SP1 regulates three genes (*Peg3*, *Ndn* and *Igf2*) as well as AACTTT_UNKNOWN (*Igf2r*, *Dlk1* and *Gnas*). We suggest these three transcription factors as candidates that may be responsible for the coregulation of this imprinting network.

Berg and colleagues [27] recently analyzed the expression levels of ten of these genes (*Cdkn1c*, *Dlk1*, *Grb10*, *Gtl2*, *H19*, *Igf2*, *Mest*, *Ndn*, *Peg3*, and *Plagl1*) in mouse long-term repopulating hematopoietic stem cells and in representative differentiated lineages. Intriguingly, they found that most of the genes were severely down regulated in differentiated cells. They noticed that their study is the first one that connected imprinted genes that are known to be associated with embryonic and early postnatal growth to the regulation of somatic stem cells. Consequently, they suggested that the balancing forces of growth-promoting paternally expressed genes and of growth-limiting maternally expressed genes may as well play a role in keeping stem cells in the delicate balance of pluripotency. Along these lines, but in the opposite direction, our above finding that the global transcription factors E12 and SP1 play key roles in the regulation of imprinted genes fits to their well-known role in cell differentiation processes [28], [29].

## Supporting Information

Table S1 **Imprinted Gene list.** The last column indicates whether the maternal (M) or paternal (P) allele is expressed. P/M means that the gene exhibits species or isoform-specific patterns of imprinting: human COPG2 and ZIM2 were reported to be paternally expressed, whereas these genes are maternally expressed in the mouse. GRB10 encodes maternally, and paternally expressed isoforms. “?” in the imprinting column indicates genes for which imprinting is not confirmed.
(DOC)

Table S2 **Enriched GO terms of biological functions for the full set of imprinted genes in human.** The table lists the annotation terms, the number of associated genes per each GO term, the ratio of genes annotated with this term relative to the total number of imprinted genes, the p-value and the fold enrichment.
(DOC)

Table S3 **Enriched GO terms of biological functions for the full set of imprinted genes in mouse.** The table lists the annotation terms, the number of associated genes per each GO term, the ratio of genes annotated with this term relative to the total number of imprinted genes, the p-value and the fold enrichment.
(DOC)

Table S4 **Enriched GO terms of biological functions for the maternally expressed genes in human and mouse.** The table lists the annotation terms, the number of associated genes per each GO term, the ratio of genes annotated with this term relative to the total number of maternally expressed genes, the p-value and the fold enrichment.
(DOC)

Table S5 **Enriched GO terms of biological functions for the paternally expressed genes in Human.** The table lists the annotation terms, the number of associated genes per each GO term, the ratio of genes annotated with this term relative to the total number of paternally expressed genes, the p-value and the fold enrichment.
(DOC)

Table S6 **The most specific enriched GO terms of biological functions for the paternally expressed genes in human.** The table lists the annotation terms, the number of the associated genes per each GO term, percentage of the involved genes to the study genes, the p-value, gene names and the fold enrichment.
(DOC)

Table S7 **The enriched Transcription factor target (TFT) families for the full set of imprinted genes in human according to the MSigDB database at significance level 0.01.** M and P are the numbers of associated maternally and paternally expressed genes respectively.
(DOC)

Table S8 **The enriched Transcription factor target (TFT) families for the full set of imprinted genes in mouse according to the MSigDB database at significance level 0.01.** M and P are the numbers of associated maternally and paternally expressed genes respectively.
(DOC)

Figure S1 **Heat map for the enriched transcription factor targets in the full set of imprinted genes in human (a) and mouse (b) at p-value of 0.01.** Marked in red and blue in the top line are the maternally and paternally expressed genes, respectively.
(TIFF)
