# Supplementary material for: Cellular Functions of Genetically Imprinted Genes in Human and Mouse as Annotated in the Gene Ontology
Source: PLoS One. 2012 Nov 30;7(11):e50285. doi: 10.1371/journal.pone.0050285 (PMC3511506; doi:10.1371/journal.pone.0050285)
Supplement: Table S1 — Imprinted Gene list. The last column indicates whether the maternal (M) or paternal (P) allele is expressed. P/M means that the gene exhibits species or isoform-specific patterns of imprinting: human COPG2 and ZIM2 were reported to be paternally expressed, whereas these genes are maternally expressed in the mouse. GRB10 encodes maternally, and paternally expressed isoforms. “?” in the imprinting column indicates genes for which imprinting is not confirmed. (DOC) [file pone.0050285.s001.doc]

**Supplementary Table 1**.

| **Gene Name** | **Description** | **Imprinting** | | **Expressed  Allele** |
| --- | --- | --- | --- | --- |
| **Human (Mouse)** |  | **Human** | **Mouse** |  |
| ASB4 (Asb4) | ankyrin repeat and SOCS box-containing 4 | ? | Y | M |
| ASCL2 (Ascl2) | achaete-scute complex homolog 2 (Drosophila) | ? | Y | M |
| ATP10A (Atp10a) | ATPase, class V, type 10A | Y | ? | M |
| BEGAIN (Begain) | brain-enriched guanylate kinase-associated homolog (rat) | ? | Y | P |
| Blcap | bladder cancer associated protein | Y | Y | M |
| C15ORF2 | chromosome 15 open reading frame 2 | Y | (no ortholog) | ? |
| CALCR (Calcr) | calcitonin receptor | ? | Y | M |
| CDKN1C (Cdkn1c) | cyclin-dependent kinase inhibitor 1C (p57, Kip2) | Y | Y | M |
| COMMD1 (Commd1) | copper metabolism (Murr1) domain containing 1 | N | Y | M |
| COPG2 (Copg2) | coatomer protein complex, subunit gamma 2 | Y | Y | P/M |
| CPA4 (Cpa4) | carboxypeptidase A4 | Y | ? | M |
| DIO3 (Dio3) | deiodinase, iodothyronine, type III | ? | Y | P |
| DLGAP2 (Dlgap2) | discs, large (Drosophila) homolog-associated protein 2 | Y | ? | P |
| DLK1 (Dlk1) | delta-like 1 homolog (Drosophila) | Y | Y | P |
| DLX5 (Dlx5) | distal-less homeobox 5 | Y | ? | M |
| GNAS (Gnas) | GNAS complex locus | Y | Y | M |
| GRB10 (Grb10) | growth factor receptor-bound protein 10 | Y | Y | P/M |
| H13 | histocompatibility 13 | ? | Y | M |
| HTR2A (Htr2a) | 5-hydroxytryptamine (serotonin) receptor 2A | ? | Y | M |
| IGF2 (Igf2) | insulin-like growth factor 2 | Y | Y | P |
| IGF2R (Igf2r) | insulin-like growth factor 2 receptor | N | Y | M |
| IMPACT (Impact) | Impact homolog (mouse) | no ortholog | Y | P |
| INPP5F (Inpp5f) | inositol polyphosphate-5-phosphatase F | ? | Y | P |
| INS (Ins2) | insulin 2 | Y | Y | P |
| KCNQ1(Kcnq1) | potassium voltage-gated channel, KQT-like subfamily, member 1 | Y | Y | M |
| KLF14 (Klf14) | Kruppel-like factor 14 | Y | Y | M |
| KCNK9 (Kcnk9) | potassium channel, subfamily K, member 9 | Y | Y | M |
| L3MBTL (L3mbtl) | lethal(3)malignant brain tumor-like protein-like | Y | N | P |
| LRRTM1 (Lrrtm1) | leucine rich repeat transmembrane neuronal 1 | Y | N | P |
| MAGEL2 (Magel2) | MAGE-like 2 | Y | Y | P |
| MCTS2 (Mcts2) | malignant T cell amplified sequence 2 | Y | Y | P |
| MEST (Mest, Peg1) | mesoderm specific transcript homolog (mouse) | Y | Y | P |
| MKRN3 (Mkrn3) | makorin ring finger protein 3 | Y | Y | P |
| NAP1L5 (Nap1l5) | nucleosome assembly protein 1-like 5 | Y | Y | P |
| NDN (Ndn) | necdin homolog (mouse) | Y | Y | P |
| NNAT (Nnat) | neuronatin | Y | Y | P |
| PEG3 (Peg3) | paternally expressed 3; PEG3 antisense RNA (non-protein coding); zinc finger, imprinted 2 | Y | Y | P |
| PEG10 (Peg10) | paternally expressed 10 | Y | Y | P |
| (Peg12) | paternally expressed 12 | no ortholog | Y | P |
| PHLDA2 (Phlda2) | pleckstrin homology-like domain, family A, member 2 | Y | Y | M |
| PLAGL1 (Plagl1) | pleiomorphic adenoma gene-like 1 | Y | ? | P |
| PPP1R9A (Ppp1r9a) | protein phosphatase 1, regulatory (inhibitor) subunit 9A | Y | Y | M |
| PRIM2 (Prim2) | primase, DNA, polypeptide 2 (58kDa) | Y | ? | M |
| RASGRF1 (Rasgrf1) | Ras protein-specific guanine nucleotide-releasing factor 1 | ? | Y | P |
| SGCE (Sgce) | sarcoglycan, epsilon | Y | Y | P |
| SLC22A18 (Slc22a18) | solute carrier family 22, member 18 | Y | Y | M |
| SLC22A2 (Slc22a2) | solute carrier family 22 (organic cation transporter), member 2 | ? | Y | M |
| SLC22A3 (Slc22a3) | solute carrier family 22 (extraneuronal monoamine transporter), member 3 | ? | Y | M |
| SLC38A4 (Slc38a4) | solute carrier family 38, member 4 | ? | Y | P |
| SNURF-SNRPN | small nuclear ribonucleoprotein polypeptide N; SNRPN upstream reading frame | Y | Y | P |
| TCEB3C | transcription elongation factor B polypeptide 3C-like; | Y | N | M |
| TFPI2 (Tfpi2) | tissue factor pathway inhibitor 2 | Y | Y | M |
| TP73 (Trp73) | tumor protein p73 | Y | ? | M |
| TRPM5 (Trpm5) | transient receptor potential cation channel, subfamily M, member 5 | ? | N | P |
| UBE3A (Ube3a) | ubiquitin protein ligase E3A | Y | Y | M |
| USP29 (Usp29) | ubiquitin specific peptidase 29 | ? | Y | P |
| WT1-Alt transcript (Wt1) | Wilms tumor 1 | Y | ? | P |
| (Zim1) | zinc finger, imprinted 1 | no ortholog | Y | M |
| ZIM2 (Zim2) | paternally expressed 3; PEG3 antisense RNA (non-protein coding); zinc finger, imprinted 2 | Y | Y | P/M |
| ZIM3 (Zim3) | zinc finger, imprinted 3 | ? | Y | M |
| ZNF264 (Zfp264) | zinc finger protein 264 | ? | Y | P |
| ZNF331 | zinc finger protein 331 | Y | ? | M |
| ZNF597 (Zfp597) | zinc finger protein 597 | Y | ? | M |
