# Supplementary material for: Cellular Functions of Genetically Imprinted Genes in Human and Mouse as Annotated in the Gene Ontology
Source: PLoS One. 2012 Nov 30;7(11):e50285. doi: 10.1371/journal.pone.0050285 (PMC3511506; doi:10.1371/journal.pone.0050285)
Supplement: Table S2 — Enriched GO terms of biological functions for the full set of imprinted genes in human. The table lists the annotation terms, the number of associated genes per each GO term, the ratio of genes annotated with this term relative to the total number of imprinted genes, the p-value and the fold enrichment. (DOC) [file pone.0050285.s002.doc]

**Supplementary Table 2**.

| term | count | % | p-value | fold enrichment |
| --- | --- | --- | --- | --- |
| GO:0048731~system development | 15 | 39.47 | 5.55E-04 | 2.60 |
| GO:0032502~developmental process | 17 | 44.74 | 1.22E-03 | 2.18 |
| GO:0048856~anatomical structure development | 15 | 39.47 | 1.28E-03 | 2.39 |
| GO:0007275~multicellular organismal development | 16 | 42.11 | 1.42E-03 | 2.25 |
| GO:0032501~multicellular organismal process | 20 | 52.63 | 1.68E-03 | 1.88 |
| GO:0030154~cell differentiation | 11 | 28.95 | 4.08E-03 | 2.71 |
| GO:0048869~cellular developmental process | 11 | 28.95 | 5.50E-03 | 2.60 |
| GO:0048513~organ development | 11 | 28.95 | 6.27E-03 | 2.55 |
| GO:0006355~regulation of transcription, DNA-dependent | 11 | 28.95 | 7.22E-03 | 2.50 |
| GO:0051252~regulation of RNA metabolic process | 11 | 28.95 | 8.44E-03 | 2.45 |
| GO:0019219~regulation of nucleobase, nucleoside,  nucleotide and nucleic acid metabolic process | 14 | 36.84 | 1.05E-02 | 2.01 |
| GO:0051171~regulation of nitrogen compound metabolic process | 14 | 36.84 | 1.13E-02 | 1.99 |
| GO:0010468~regulation of gene expression | 14 | 36.84 | 1.19E-02 | 1.98 |
| GO:0009791~post-embryonic development | 3 | 7.89 | 1.33E-02 | 16.57 |
| GO:0045449~regulation of transcription | 13 | 34.21 | 1.48E-02 | 2.02 |
| GO:0009987~cellular process | 32 | 84.21 | 1.50E-02 | 1.22 |
| GO:0031326~regulation of cellular biosynthetic process | 14 | 36.84 | 1.54E-02 | 1.92 |
| GO:0009889~regulation of biosynthetic process | 14 | 36.84 | 1.62E-02 | 1.90 |
| GO:0033673~negative regulation of kinase activity | 3 | 7.89 | 1.98E-02 | 13.44 |
| GO:0030182~neuron differentiation | 5 | 13.16 | 2.03E-02 | 4.60 |
| GO:0006139~nucleobase, nucleoside, nucleotide and nucleic  acid metabolic process | 15 | 39.47 | 2.10E-02 | 1.77 |
| GO:0051348~negative regulation of transferase activity | 3 | 7.89 | 2.23E-02 | 12.60 |
| GO:0006350~transcription | 11 | 28.95 | 2.27E-02 | 2.11 |
| GO:0006349~regulation of gene expression by genetic imprinting | 2 | 5.26 | 2.38E-02 | 80.66 |
| GO:0007050~cell cycle arrest | 3 | 7.89 | 2.54E-02 | 11.75 |
| GO:0008219~cell death | 6 | 15.79 | 2.76E-02 | 3.37 |
| GO:0010556~regulation of macromolecule biosynthetic process | 13 | 34.21 | 2.78E-02 | 1.85 |
| GO:0016265~death | 6 | 15.79 | 2.84E-02 | 3.34 |
| GO:0060255~regulation of macromolecule metabolic process | 14 | 36.84 | 3.40E-02 | 1.73 |
| GO:0019222~regulation of metabolic process | 15 | 39.47 | 3.44E-02 | 1.67 |
| GO:0010646~regulation of cell communication | 7 | 18.42 | 3.56E-02 | 2.72 |
| GO:0080090~regulation of primary metabolic process | 14 | 36.84 | 3.66E-02 | 1.72 |
| GO:0034641~cellular nitrogen compound metabolic process | 15 | 39.47 | 3.83E-02 | 1.65 |
| GO:0048523~negative regulation of cellular process | 9 | 23.68 | 3.99E-02 | 2.19 |
| GO:0032870~cellular response to hormone stimulus | 3 | 7.89 | 4.06E-02 | 9.10 |
| GO:0001501~skeletal system development | 4 | 10.53 | 4.08E-02 | 5.06 |
| GO:0051051~negative regulation of transport | 3 | 7.89 | 4.17E-02 | 8.96 |
| GO:0007399~nervous system development | 7 | 18.42 | 4.33E-02 | 2.59 |
| GO:0048699~generation of neurons | 5 | 13.16 | 4.41E-02 | 3.61 |
| GO:0046626~regulation of insulin receptor signaling pathway | 2 | 5.26 | 4.48E-02 | 42.45 |
| GO:0009887~organ morphogenesis | 5 | 13.16 | 4.53E-02 | 3.58 |
| GO:0048666~neuron development | 4 | 10.53 | 4.75E-02 | 4.76 |
| GO:0006807~nitrogen compound metabolic process | 15 | 39.47 | 4.81E-02 | 1.60 |
