# Supplementary material for: Cellular Functions of Genetically Imprinted Genes in Human and Mouse as Annotated in the Gene Ontology
Source: PLoS One. 2012 Nov 30;7(11):e50285. doi: 10.1371/journal.pone.0050285 (PMC3511506; doi:10.1371/journal.pone.0050285)
Supplement: Table S3 — Enriched GO terms of biological functions for the full set of imprinted genes in mouse. The table lists the annotation terms, the number of associated genes per each GO term, the ratio of genes annotated with this term relative to the total number of imprinted genes, the p-value and the fold enrichment. (DOC) [file pone.0050285.s003.doc]

**Supplementary Table 3**.

| term | count | % | p-value | fold enrichment |
| --- | --- | --- | --- | --- |
| GO:0043085~positive regulation of catalytic activity | 5 | 10.42 | 6.03E-03 | 6.64 |
| GO:0044093~positive regulation of molecular function | 5 | 10.42 | 1.05E-02 | 5.67 |
| GO:0032502~developmental process | 15 | 31.25 | 1.42E-02 | 1.90 |
| GO:0007275~multicellular organismal development | 14 | 29.17 | 1.73E-02 | 1.93 |
| GO:0048731~system development | 12 | 25.00 | 2.11E-02 | 2.05 |
| GO:0042493~response to drug | 3 | 6.25 | 2.54E-02 | 11.82 |
| GO:0048856~anatomical structure development | 12 | 25.00 | 3.33E-02 | 1.92 |
| GO:0065008~regulation of biological quality | 8 | 16.67 | 3.52E-02 | 2.47 |
| GO:0015695~organic cation transport | 2 | 4.17 | 3.60E-02 | 53.35 |
| GO:0042221~response to chemical stimulus | 7 | 14.58 | 3.62E-02 | 2.74 |
| GO:0050790~regulation of catalytic activity | 5 | 10.42 | 4.00E-02 | 3.75 |
| GO:0007242~intracellular signaling cascade | 7 | 14.58 | 4.12E-02 | 2.65 |
| GO:0019932~second-messenger-mediated signaling | 3 | 6.25 | 4.34E-02 | 8.82 |
| GO:0042325~regulation of phosphorylation | 4 | 8.33 | 4.77E-02 | 4.78 |
| GO:0006811~ion transport | 6 | 12.50 | 4.80E-02 | 2.92 |
| GO:0048666~neuron development | 4 | 8.33 | 4.85E-02 | 4.75 |
