# Supplementary material for: Cellular Functions of Genetically Imprinted Genes in Human and Mouse as Annotated in the Gene Ontology
Source: PLoS One. 2012 Nov 30;7(11):e50285. doi: 10.1371/journal.pone.0050285 (PMC3511506; doi:10.1371/journal.pone.0050285)
Supplement: Table S4 — Enriched GO terms of biological functions for the maternally expressed genes in human and mouse. The table lists the annotation terms, the number of associated genes per each GO term, the ratio of genes annotated with this term relative to the total number of maternally expressed genes, the p-value and the fold enrichment. (DOC) [file pone.0050285.s004.doc]

**Supplementary Table 4**.

| Human | | | | | |
| --- | --- | --- | --- | --- | --- |
| Term | Count | % | p-value | Genes | Fold Enrichment |
| GO:0045669~positive regulation of osteoblast differentiation | 2 | 10.53 | 3.14E-02 | DLX5, GNAS | 59.44 |
| GO:0009887~organ morphogenesis | 4 | 21.05 | 3.31E-02 | DLX5, GNAS, TP73, PHLDA2 | 5.27 |
| GO:0007399~nervous system development | 5 | 26.32 | 4.50E-02 | CDKN1C, PPP1R9A, UBE3A, DLX5, TP73 | 3.41 |
| Mouse | | | | | |
| GO:0042493~response to drug | 3 | 12.50 | 6.69E-03 | Slc22a18, Gnas, Htr2a | 23.08 |
| GO:0019932~second-messenger-mediated signaling | 3 | 12.50 | 1.18E-02 | Calcr, Gnas, Htrh2a | 17.21 |
| GO:0006811~ion transport | 5 | 20.83 | 1.59E-02 | Kcnk9, Slc22a18, Slc22a3, Kcnq1, Slc22a2 | 4.75 |
| GO:0007189~activation of adenylate cyclase activity  by G-protein signaling pathway | 2 | 8.33 | 4.41E-02 | Calcr, Gnas | 42.32 |
| GO:0010579~positive regulation of adenylate cyclase  activity by G-protein signaling pathway | 2 | 8.33 | 4.41E-02 | Calcr, Gnas | 42.32 |
| GO:0010578~regulation of adenylate cyclase activity  involved in G-protein signaling | 2 | 8.33 | 4.41E-02 | Calcr, GnasS | 42.32 |
| GO:0015695~organic cation transport | 2 | 8.33 | 1.81E-02 | Slc22a3, Slc22a2 | 99.17 |
| GO:0055085~transmembrane transport | 4 | 16.67 | 2.55E-02 | Slc22a18, Slc22a3,  Kcnq1, Slc22a2 | 5.89 |
| GO:0006812~cation transport | 4 | 16.67 | 3.41E-02 | Kcnk9, Slc22a3, Kcnq1, Slc22a2 | 5.26 |
| GO:0006810~transport | 8 | 33.33 | 3.49E-02 | Copg2, Kcnk9, Slc22a18, Ube3a, Igf2r, Slc22a3, Kcnq1, Slc22a2 | 2.31 |
| GO:0007242~intracellular signaling cascade | 5 | 20.83 | 3.61E-02 | Calcr, Ube3a, Gnas, Asb4, Htr2a | 3.70 |
| GO:0051234~establishment of localization | 8 | 33.33 | 3.61E-02 | Copg2, Kcnk9, Slc22a18, Ube3a, Igf2r, Slc22a3, Kcnq1, Slc22a2 | 2.30 |
| GO:0019222~regulation of metabolic process | 9 | 37.50 | 3.86E-02 | Calcr, Cdkn1c, Ascl2, Ube3a, Zim1, Gnas, Klf14, Kcnq1, Htr2a | 2.07 |
