# Supplementary material for: Cellular Functions of Genetically Imprinted Genes in Human and Mouse as Annotated in the Gene Ontology
Source: PLoS One. 2012 Nov 30;7(11):e50285. doi: 10.1371/journal.pone.0050285 (PMC3511506; doi:10.1371/journal.pone.0050285)
Supplement: Table S5 — Enriched GO terms of biological functions for the paternally expressed genes in Human. The table lists the annotation terms, the number of associated genes per each GO term, the ratio of genes annotated with this term relative to the total number of paternally expressed genes, the p-value and the fold enrichment. (DOC) [file pone.0050285.s005.doc]

**Supplementary Table 5.**

| Term | Count | % | p-value | Genes | Fold Enrichment |
| --- | --- | --- | --- | --- | --- |
| GO:0048731~system development | 8 | 47.06 | 2.25E-03 | NDN, INS, NNAT, L3MBTL, IGF2, SGCE, DLK1, WT1, MEST | 3.46 |
| GO:0032502~developmental process | 9 | 52.94 | 2.62E-03 | PEG10, NDN, INS, NNAT, L3MBTL, IGF2, SGCE, DLK1, WT1, MEST | 2.88 |
| GO:0048856~anatomical structure development | 8 | 47.06 | 3.65E-03 | NDN, INS, NNAT, L3MBTL, IGF2, SGCE, DLK1, WT1, MEST | 3.19 |
| GO:0007275~multicellular organismal development | 8 | 47.06 | 7.59E-03 | NDN, INS, NNAT, L3MBTL, IGF2, SGCE, DLK1, WT1, MEST | 2.82 |
| GO:0030154~cell differentiation | 6 | 35.29 | 1.20E-02 | PEG10, NDN, INS, NNAT, IGF2, DLK1, WT1 | 3.70 |
| GO:0048869~cellular developmental process | 6 | 35.29 | 1.43E-02 | PEG10, NDN, INS, NNAT, IGF2, DLK1, WT1 | 3.55 |
| GO:0048513~organ development | 6 | 35.29 | 1.54E-02 | INS, NNAT, L3MBTL, IGF2, SGCE, WT1, MEST | 3.48 |
| GO:0006355~regulation of transcription, DNA-dependent | 6 | 35.29 | 1.68E-02 | PLAGL1, NDN, INS, L3MBTL, IGF2, WT1, ZIM2, PEG3 | 3.41 |
| GO:0051252~regulation of RNA metabolic process | 6 | 35.29 | 1.84E-02 | PLAGL1, NDN, INS, L3MBTL, IGF2, WT1, ZIM2, PEG3 | 3.34 |
| GO:0032501~multicellular organismal process | 9 | 52.94 | 1.94E-02 | NDN, INS, DLGAP2, NNAT, L3MBTL, IGF2, SGCE, DLK1, WT1, MEST | 2.12 |
