# Supplementary material for: Cellular Functions of Genetically Imprinted Genes in Human and Mouse as Annotated in the Gene Ontology
Source: PLoS One. 2012 Nov 30;7(11):e50285. doi: 10.1371/journal.pone.0050285 (PMC3511506; doi:10.1371/journal.pone.0050285)
Supplement: Table S6 — The most specific enriched GO terms of biological functions for the paternally expressed genes in human. The table lists the annotation terms, the number of the associated genes per each GO term, percentage of the involved genes to the study genes, the p-value, gene names and the fold enrichment. (DOC) [file pone.0050285.s006.doc]

**Supplementary Table 6**.

| Term | Count | % | p-value | Genes | Fold Enrichment |
| --- | --- | --- | --- | --- | --- |
| GO:0006355~regulation of transcription,  DNA-dependent | 6 | 35.29 | 1.99E-02 | PLAGL1, L3MBTL,  IGF2, WT1, ZIM2, PEG3 | 3.27 |
| GO:0051252~regulation of RNA  metabolic process | 6 | 35.29 | 2.18E-02 | PLAGL1, L3MBTL,  IGF2, WT1, ZIM2, PEG3 | 3.20 |
