# Supplementary material for: Cellular Functions of Genetically Imprinted Genes in Human and Mouse as Annotated in the Gene Ontology
Source: PLoS One. 2012 Nov 30;7(11):e50285. doi: 10.1371/journal.pone.0050285 (PMC3511506; doi:10.1371/journal.pone.0050285)
Supplement: Table S7 — The enriched Transcription factor target (TFT) families for the full set of imprinted genes in human according to the MSigDB database at significance level 0.01. M and P are the numbers of associated maternally and paternally expressed genes respectively. (DOC) [file pone.0050285.s007.doc]

**Supplement Table 7.**

| TFT Family | Genes | Count | P-Value | M | P |
| --- | --- | --- | --- | --- | --- |
| hsa_GGGCGGR_V$SP1_Q6 | TFPI2 UBE3A L3MBTL KLF14 NNAT  NDN WT1 ATP10A PEG3 PHLDA2 IGF2 | 11 | 1.00E-04 | 5 | 6 |
| hsa_V$NKX3A_01 | NAP1L5 LRRTM1 NNAT CPA4 | 4 | 4.00E-04 | 1 | 3 |
| hsa_V$FOXO4_02 | GNAS NAP1L5 CDKN1C NNAT | 4 | 4.00E-04 | 2 | 2 |
| hsa_CAGGTG_V$E12_Q6 | GNAS CDKN1C NNAT WT1 KCNQ1  CPA4 MAGEL2 IGF2 DLK1 | 9 | 4.00E-04 | 4 | 5 |
| hsa_TGGNNNNNNKCCAR_UNKNOWN | KLF14 LRRTM1 NNAT BLCAP MAGEL2 | 5 | 4.00E-04 | 2 | 3 |
| hsa_TGGAAA_V$NFAT_Q4_01 | GNAS NAP1L5 KLF14 LRRTM1 NNAT  GRB10 BLCAP INS | 8 | 4.00E-04 | 3 | 5 |
| hsa_RTAAACA_V$FREAC2_01 | NAP1L5 L3MBTL CDKN1C KLF14  MKRN3 BLCAP | 6 | 5.00E-04 | 3 | 3 |
| hsa_V$MEF2_01 | GNAS KCNK9 UBE3A | 3 | 1.40E-03 | 3 | 0 |
| hsa_TGACAGNY_V$MEIS1_01 | UBE3A KLF14 NNAT GRB10 CPA4 | 5 | 2.10E-03 | 3 | 2 |
| hsa_AACTTT_UNKNOWN | GNAS NAP1L5 PLAGL1 KLF14 LRRTM1  BLCAP DLK1 | 7 | 2.10E-03 | 3 | 4 |
| hsa_V$NCX_01 | GNAS PLAGL1 PEG3 | 3 | 2.10E-03 | 1 | 2 |
| hsa_V$YY1_Q6 | SNURF UBE3A PEG3 | 3 | 3.80E-03 | 1 | 2 |
| hsa_V$HSF2_01 | KCNK9 NNAT KCNQ1 | 3 | 3.80E-03 | 2 | 1 |
| hsa_V$GCM_Q2 | GNAS L3MBTL LRRTM1 | 3 | 3.80E-03 | 1 | 2 |
| hsa_V$E12_Q6 | GNAS CPA4 IGF2 | 3 | 3.80E-03 | 2 | 1 |
| hsa_V$GATA1_03 | UBE3A KLF14 IGF2 | 3 | 3.80E-03 | 2 | 1 |
| hsa_V$E2A_Q2 | GNAS MKRN3 IGF2 | 3 | 3.80E-03 | 1 | 2 |
| hsa_V$SREBP_Q3 | UBE3A BLCAP PEG3 | 3 | 3.80E-03 | 2 | 1 |
| hsa_TGANTCA_V$AP1_C | TFPI2 UBE3A SLC22A18 CPA4  PHLDA2 | 5 | 4.80E-03 | 5 | 0 |
| hsa_TTCYRGAA_UNKNOWN | NNAT GRB10 KCNQ1 | 3 | 5.10E-03 | 1 | 2 |
| hsa_YAATNANRNNNCAG_UNKNOWN | GNAS NAP1L5 | 2 | 5.40E-03 | 1 | 1 |
| hsa_CTGCAGY_UNKNOWN | SNURF LRRTM1 NNAT CPA4 | 4 | 7.30E-03 | 1 | 3 |
| hsa_TATAAA_V$TATA_01 | KLF14 BLCAP CPA4 PEG3 INS | 5 | 7.80E-03 | 3 | 2 |
| hsa_TTGTTT_V$FOXO4_01 | GNAS NAP1L5 UBE3A CDKN1C  LRRTM1 BLCAP | 6 | 7.80E-03 | 4 | 2 |
| hsa_V$SREBP1_02 | GNAS CPA4 | 2 | 7.80E-03 | 2 | 0 |
