# Supplementary material for: Cellular Functions of Genetically Imprinted Genes in Human and Mouse as Annotated in the Gene Ontology
Source: PLoS One. 2012 Nov 30;7(11):e50285. doi: 10.1371/journal.pone.0050285 (PMC3511506; doi:10.1371/journal.pone.0050285)
Supplement: Table S8 — The enriched Transcription factor target (TFT) families for the full set of imprinted genes in mouse according to the MSigDB database at significance level 0.01. M and P are the numbers of associated maternally and paternally expressed genes respectively. (DOC) [file pone.0050285.s008.doc]

**Supplement Table 8.**

| TFT Family | Genes | Count | P-Value | M | P |
| --- | --- | --- | --- | --- | --- |
| mmu_AACTTT_UNKNOWN | Copg2 Gnas Igf2r Klf14 Nap1l5 Blcap Dlk1 Htr2a Asb4 | 9 | 8.73E-05 | 7 | 2 |
| mmu_V$MEF2_01 | Gnas Ascl2 Kcnk9 Ube3a | 4 | 8.73E-05 | 4 | 0 |
| mmu_TTGTTT_V$FOXO4_01 | Gnas Slc22a2 Ascl2 Nap1l5 Ube3a Blcap Slc38a4 Cdkn1c | 8 | 3.00E-04 | 6 | 2 |
| mmu_V$FOXO4_02 | Gnas Nnat Nap1l5 Cdkn1c | 4 | 3.00E-04 | 2 | 2 |
| mmu_TGGAAA_V$NFAT_Q4_01 | Gnas Calcr Nnat Klf14 Nap1l5 Blcap Grb10 Inpp5f | 8 | 3.00E-04 | 5 | 3 |
| mmu_GGGCGGR_V$SP1_Q6 | Ndn Nnat Tfpi2 Klf14 Ube3a Dio3 Phlda2 Peg3 Igf2 | 9 | 4.00E-04 | 4 | 5 |
| mmu_TGANTCA_V$AP1_C | Slc22a2 Ascl2 Tfpi2 Ube3a Slc22a18 Phlda2 | 6 | 6.00E-04 | 6 | 0 |
| mmu_CAGGTG_V$E12_Q6 | Kcnq1 Gnas Magel2 Nnat Dio3 Dlk1 Cdkn1c Igf2 | 8 | 7.00E-04 | 3 | 5 |
| mmu_TATAAA_V$TATA_01 | Klf14 Dio3 Blcap Slc38a4 Asb4 Peg3 | 6 | 9.00E-04 | 3 | 3 |
| mmu_TGGNNNNNNKCCAR_UNKNOWN | Magel2 Nnat Klf14 Blcap | 4 | 9.00E-04 | 2 | 2 |
| mmu_RTAAACA_V$FREAC2_01 | Mkrn3 Klf14 Nap1l5 Blcap Cdkn1c | 5 | 1.00E-03 | 3 | 2 |
| mmu_TCANNTGAY_V$SREBP1_01 | Rasgrf1 Igf2r Nap1l5 Blcap | 4 | 1.00E-03 | 2 | 2 |
| mmu_V$SRY_01 | Ascl2 Slc38a4 Cdkn1c | 3 | 1.60E-03 | 2 | 1 |
| mmu_GGGAGGRR_V$MAZ_Q6 | Gnas Calcr Ascl2 Nnat Tfpi2 Igf2 Inpp5f | 7 | 1.60E-03 | 4 | 3 |
| mmu_V$NKX3A_01 | Nnat Nap1l5 Asb4 | 3 | 1.60E-03 | 1 | 2 |
| mmu_V$HNF1_C | Klf14 Ube3a Asb4 | 3 | 1.80E-03 | 3 | 0 |
| mmu_V$E2A_Q2 | Gnas Mkrn3 Igf2 | 3 | 1.80E-03 | 1 | 2 |
| mmu_V$DBP_Q6 | Kcnq1 Calcr Nap1l5 | 3 | 1.80E-03 | 2 | 1 |
| mmu_V$GATA1_03 | Klf14 Ube3a Igf2 | 3 | 1.80E-03 | 2 | 1 |
| mmu_V$HSF2_01 | Kcnq1 Nnat Kcnk9 | 3 | 1.80E-03 | 2 | 1 |
| mmu_V$SREBP_Q3 | Ube3a Blcap Peg3 | 3 | 1.80E-03 | 2 | 1 |
| mmu_V$PXR_Q2 | Copg2 Ndn Asb4 | 3 | 2.20E-03 | 2 | 1 |
| mmu_V$IK2_01 | Ascl2 Nnat Asb4 | 3 | 2.40E-03 | 2 | 1 |
| mmu_YWATTWNNRGCT_UNKNOWN | Rasgrf1 Klf14 | 2 | 2.40E-03 | 1 | 1 |
| mmu_TTANWNANTGGM_UNKNOWN | Klf14 Asb4 | 2 | 2.40E-03 | 2 | 0 |
| mmu_YAATNANRNNNCAG_UNKNOWN | Gnas Nap1l5 | 2 | 2.40E-03 | 1 | 1 |
| mmu_CTTTGT_V$LEF1_Q2 | Calcr Mkrn3 Igf2r Nnat Slc38a4 Cdkn1c | 6 | 2.50E-03 | 3 | 3 |
| mmu_TGCCAAR_V$NF1_Q6 | Copg2 Magel2 Nnat Slc38a4 | 4 | 2.50E-03 | 1 | 3 |
| mmu_TTCYRGAA_UNKNOWN | Kcnq1 Nnat Grb10 | 3 | 2.50E-03 | 2 | 1 |
| mmu_TGTTTGY_V$HNF3_Q6 | Slc22a2 Ascl2 Slc38a4 Asb4 | 4 | 2.90E-03 | 3 | 1 |
| mmu_TGACAGNY_V$MEIS1_01 | Nnat Klf14 Ube3a Grb10 | 4 | 3.60E-03 | 3 | 1 |
| mmu_V$CDP_01 | Calcr Klf14 | 2 | 4.20E-03 | 2 | 0 |
| mmu_RGAANNTTC_V$HSF1_01 | Kcnq1 Gnas Nnat | 3 | 6.30E-03 | 2 | 1 |
| mmu_V$GATA2_01 | Gnas Blcap | 2 | 6.50E-03 | 2 | 0 |
| mmu_CTTTAAR_UNKNOWN | Igf2r Phlda2 Peg3 Inpp5f | 4 | 6.70E-03 | 2 | 2 |
| mmu_V$MEIS1AHOXA9_01 | Klf14 Nap1l5 | 2 | 6.80E-03 | 1 | 1 |
| mmu_YGCANTGCR_UNKNOWN | Ascl2 Nnat | 2 | 7.00E-03 | 1 | 1 |
| mmu_V$GRE_C | Nnat Ube3a | 2 | 7.40E-03 | 1 | 1 |
| mmu_RGAGGAARY_V$PU1_Q6 | Kcnq1 Ndn Inpp5f | 3 | 7.90E-03 | 1 | 2 |
| mmu_V$MEIS1BHOXA9_01 | Rasgrf1 Klf14 | 2 | 8.60E-03 | 1 | 1 |
