# Supplementary figures and images for: Cellular Functions of Genetically Imprinted Genes in Human and Mouse as Annotated in the Gene Ontology
Source: PLoS One. 2012 Nov 30;7(11):e50285. doi: 10.1371/journal.pone.0050285 (PMC3511506; doi:10.1371/journal.pone.0050285)

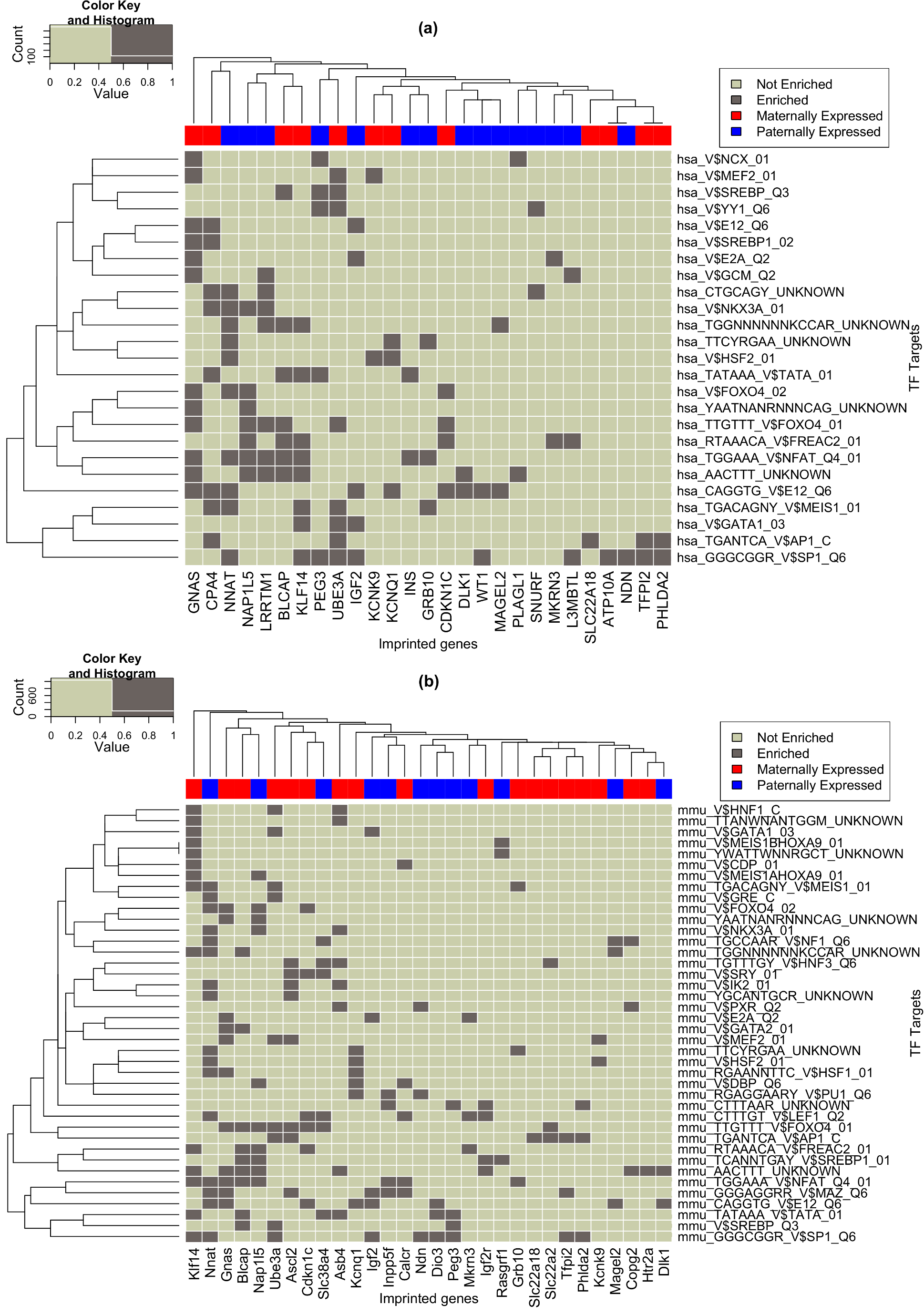

Supplement: Figure S1 — Heat map for the enriched transcription factor targets in the full set of imprinted genes in human (a) and mouse (b) at p-value of 0.01. Marked in red and blue in the top line are the maternally and paternally expressed genes, respectively. (TIFF) [file pone.0050285.s009.tiff]
